# Extracorporeal cardiopulmonary resuscitation in out-of-hospital cardiac arrest – current status

**DOI:** 10.1097/MCC.0000000000001102

**Published:** 2023-10-20

**Authors:** Martje M. Suverein, Jos G. Maessen, Marcel C.G. van de Poll

**Affiliations:** a Department of Intensive Care, Maastricht University Medical Center; b Department of Cardiothoracic Surgery, Maastricht University Medical Center Cardiovascular Research Institute Maastricht; c Department of Intensive Care, Maastricht University Medical Center, Maastricht, the Netherlands School for Nutrition and Translational Research in Metabolism, Maastricht University, Maastricht, The Netherlands

**Keywords:** cardiopulmonary resuscitation, extracorporeal cardiopulmonary resuscitation, extracorporeal membrane oxygenation

## Abstract

**Purpose of review:**

Extracorporeal cardiopulmonary resuscitation (ECPR) is an emerging treatment for refractory cardiac arrest. In recent years, several randomized controlled trials have been published that aimed to address the efficacy and effectiveness of ECPR for out-of-hospital cardiac arrest (OHCA). Despite the lack of high-quality evidence concerning clinical effectiveness and cost-effectiveness, ECPR is increasingly implemented throughout the world. In this review, we aim to provide an overview of the current status of ECPR for OHCA.

**Recent findings:**

Randomized controlled trials showed diverging results, largely due to differences in selection criteria and study design. Single-center studies, performed in centers with extraordinary expertise and dedication consistently achieve a low-flow time of around 60 min, but such achievements are rarely reproduced outside these centers. Strict patient selection can improve outcome but simultaneously limits the caseload. Preliminary data suggest that outcome may also be improved by avoiding hyperoxia postresuscitation.

**Summary:**

The potential of ECPR to increase survival in selected patients in highly dedicated systems seems to be proven, the question remains whether ECPR for OHCA can be widely implemented successfully and can develop into a sustainable, commonplace resource-effective treatment.

## INTRODUCTION

The incidence of EMS-treated out-of-hospital cardiac arrest (OHCA) is approximately 30–90 per 100 000 persons per year in the developed world with an overall mortality of 84-95% [1,2]. Patients presenting with a shockable rhythm have the best chance of survival, particularly when cardiac arrest is witnessed, immediate basic life support is started and defibrillation attempts are performed using a public automated external defibrillator (AED) [3]. However, when return of spontaneous circulation does not occur within 15 min of advanced circulatory life support (ACLS), the cardiac arrest is considered refractory and the probability of survival rapidly declines [4]. In these cases, the application of venoarterial extracorporeal membrane oxygenation can restore circulation and organ perfusion. This strategy is referred to as extracorporeal cardiopulmonary resuscitation (ECPR) and is increasingly used as a rescue treatment that unlocks the possibility to treat the underlying cause of cardiac arrest, with the ultimate goal to restore spontaneous circulation. During recent years much data have been published that elucidate the potential of ECPR, but also its limitations. This review aims to provide an overview of the current status of EPCR for OHCA. 

**Box 1:**
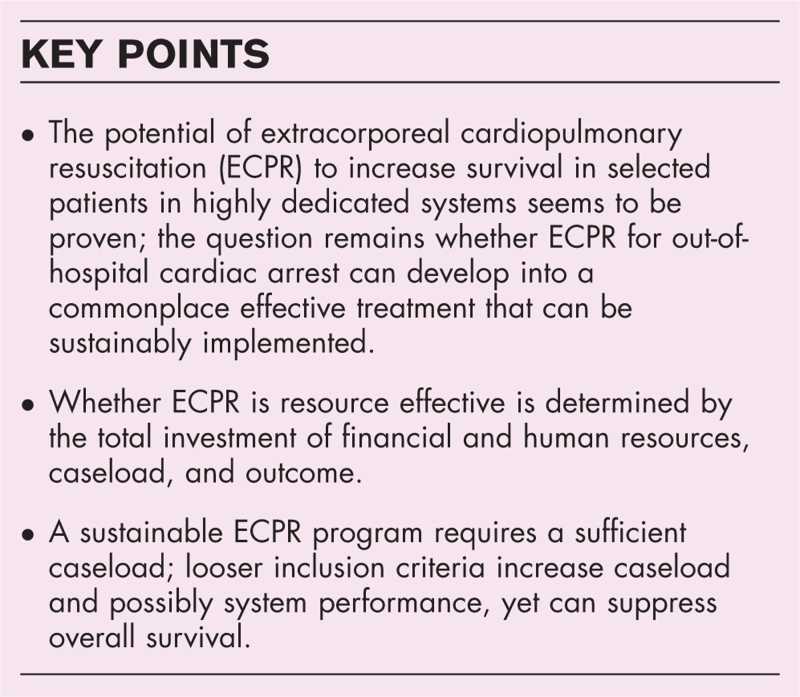
no caption available

## EFFICACY AND EFFECTIVENESS

Until 2020 all available evidence on ECPR for OHCA has been derived from observational data, particularly originating from southeast Asia, Germany, and France [5]. In 2020, the first randomized controlled trial (RCT) with a clinical endpoint addressing ECPR for OHCA was published [6^▪▪^]. The ARREST trial randomized 30 adult patients with an initial shockable rhythm, who were presented at the Emergency Department (ED) without return of spontaneous circulation (ROSC) after at least three defibrillation attempts. Patients with an unfavorable metabolic profile were excluded from ECPR despite being allocated to ECPR, but retained in the intention-to-treat analysis. This single-center study which was terminated early for overwhelmingly positive outcomes showed a 36.2 absolute risk reduction for its primary outcome, hospital survival, in favor of ECPR and demonstrated the efficacy of ECPR within a fully dedicated multidisciplinary, transmural system.

**FIGURE 1:**
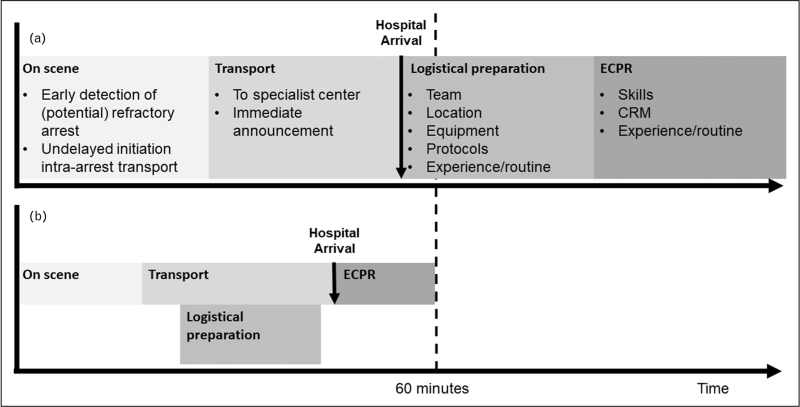
Procedure of in-hospital extracorporeal cardiopulmonary resuscitation (ECPR) for out-of-hospital cardiac arrest (OHCA). The entire procedure of ECPR for OHCA contains several consecutive steps between cardiac arrest and start of ECMO circulation. Each step of the procedure contains elements that can be optimized to create ideal conditions to perform ECPR and to limit low-flow times, ideally to less than 60 min. Transport times may be increased when a patient is transferred to an expert center, but timely announcement of an incoming ECPR candidate can facilitate the receiving center to perform logistic preparations during transport. Early activation of the process limits low-flow time but can lead to redundant ECPR system activation when patients regain ROSC during transport to the hospital. (a) Consecutive steps with points of improvement. (b) Optimal performing system.

More recently, the Prague OHCA study was published [7^▪▪^]. This single-center RCT differed from the ARREST trial in several aspects of the study design. Most importantly, patients were randomized prehospital and became eligible after 5 min of unsuccessful advanced cardiac life support. Moreover, patients with all presenting rhythms were eligible, providing that OHCA was of presumed cardiac origin. Patients were either transported to an ECPR-capable hospital using mechanical chest compression devices or remained on scene. Patients arriving at the hospital without ROSC received ECPR. The trial was discontinued after 256 patients when a prespecified between-group difference of 15% could no longer be achieved. The absolute risk reduction for the primary endpoint (survival with favorable neurology after 6 months) was 9%. The lower risk reduction in the Prague OHCA study in comparison with the ARREST trial can be ascribed to the inclusion of patients with nonshockable rhythms [8] but also to prehospital randomization and higher-than-expected survival in the control group. While prehospital randomization may obscure the “true ECPR efficacy”, it notably appreciates the fact that ECPR does not start upon hospital arrival, but at the scene of OHCA (Fig. 1). When assessing the true added impact and cost of ECPR, it is important to also consider the ECPR system initiations that prove to be redundant due to patients regaining ROSC before hospital arrival.

While the potential of ECPR to increase survival in selected patients in highly dedicated systems seems to be proven [6^▪▪^,^7^^▪▪^,^9^], the question remains whether ECPR for OHCA can develop into a commonplace effective treatment that can be sustainably implemented outside of these frontrunner centers. To assess whether an intervention, that was shown to be efficacious under ideal circumstances, is also effective in the real world, the performance of pragmatic multicenter trials is necessary. The INCEPTION trial was a trial that randomized 134 patients with an initial shockable rhythm between ECPR and CCPR in 10 Dutch cardiosurgical centers [10,11^▪▪^]. The absolute risk reduction for the primary endpoint, survival with favorable neurology after 30 days was 4%. This difference was not statistically significant. A major point of criticism that was raised concerning the external validity of the INCEPTION trial is the time from cardiac arrest to ECMO flow which was 74 min median, which is substantially longer than in the ARREST trial and the Prague OHCA study (mean 59 min and median 61 min, respectively). However, the low flow times achieved during the INCEPTION trial are comparable to those typically achieved by other large centers presenting their experience with ECPR [12,13^▪^,^14^,15^▪^,^16^–^18^], underlining the real-world representativity and external validity of the results of the INCEPTION trial.

**FIGURE 2:**
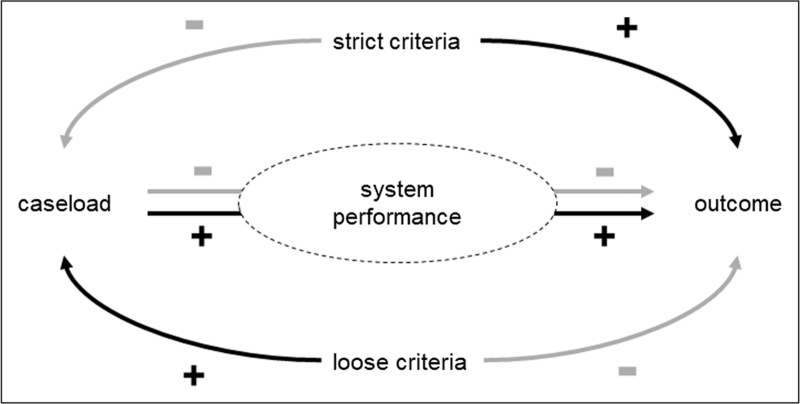
Schematic representation of some factors of extracorporeal cardiopulmonary resuscitation (ECPR) that influence system performance. While stricter inclusion criteria for ECPR results in better outcome, it also suppresses the number of treated patients which lessens a center's experience and can decrease their system performance which can eventually diminish their outcome.

ECPR for OHCA is a complex and resource-intensive therapy with an intricate interplay between caseload, patient selection, outcome, resource allocation, and cost-effectiveness (Fig. 2). Despite the ongoing broad implementation, it is still unknown what are the minimal requirements to achieve a balance between allocated resources and incremental effectiveness and whether such a “sweet spot” exists at all [19].

## COST-EFFECTIVENESS

Several recent reviews concluded that hospital-based ECPR for OHCA can be cost-effective when compared with conventional CPR. However, cost-effectiveness was mainly dependent on a high incremental effectiveness of ECPR versus conventional CPR, since the treatment costs of ECPR are substantially greater [20,21]. Hitherto, all cost-effectiveness studies on ECPR for OHCA were not performed alongside a randomized trial, meaning that only results of modeling approaches are at hand, which are fueled by observational data or expert opinion [22,23]. Other notable limitations are the fact that indirect costs, such as productivity losses, nonmedical costs, and adverse events generally have not been taken into account. The estimated incremental cost-effectiveness ratios range from 12 254 to 155 739 EUR per quality-adjusted life year [21]. The judgment of cost-effectiveness obviously also depends on the willingness-to-pay threshold that is variable amongst countries. The health economic analyses that were performed alongside the recently published RCTs are awaited to gain more reliable information about cost-effectiveness.

Apart from the substantial utilization of financial resources, ECPR may place a substantial burden on immaterial human resources [24]. The benchmark ECPR programs rely on the involvement of very small and highly dedicated teams that are on call for ECPR 24/7 [9]. The question is whether relying on such small teams is a solid basis for sustainable infrastructure and whether such personal dedication can be demanded as standard of care. Eventual resource effectiveness is determined by the total investment of financial and human resources, caseload, and outcome. Increasing the allocation of financial and human resources should lead to improved outcome in order to maintain or improve resource effectiveness. Thus, the investments to maintain a system that is continuously stand-by should be taken into account. Resource allocation should be weighed against the caseload, however, when an increased caseload comes at the cost of reduced effectiveness the resource effectiveness will go down (Fig. 2).

## PATIENT NUMBERS

A sustainable ECPR program requires a sufficient caseload. In the first place, regular exposure is needed to gain and maintain skills, and experience with complex logistics and procedures. In the second place, a substantial caseload (with an acceptable survival rate) is necessary to justify allocated resources and to increase cost-effectiveness [19]. Data from the Extracorporeal Life Support Organization have shown that the outcome of ECPR sharply declines in centers performing <9–12 cases of ECPR per year. Notably, only 44 centers reported a caseload of >6 ECPR cases per year and only 10 centers reported a caseload of >12 per year [25]. Several theoretical models have been published in the last few years that estimate the number of ECPR-eligible patients in different settings. The results vary between ≈2 and ≈130 potential ECPR candidates per one million inhabitants per year [26–28]. From the inclusion rates and adherence populations of the available prospective trials, it can be deduced that the number of ECPR procedures in patients with a truly refractory cardiac arrest and a shockable rhythm, that are performed within systems that are actively screening for these patients, lies around 1 patient per million inhabitants per month.

Obviously, the caseload can be increased when criteria are loosened. Pulmonary embolism is often suggested as an additional indication, yet prospective evidence is still lacking and a posthoc analysis of the Prague OHCA study did not show an improved outcome [29]. Increasing caseload by loosening selection criteria can potentially improve system performance, however, it will inevitably lead to a worse outcome. It may be argued that loose selection criteria may be acceptable to increase the absolute number of survivors [30], but a low clinical effectiveness may in the end become a severe threat to the institutional support for ECPR, not only because it impairs resource-effectiveness, but also since ECPR is an arduous event for ED nursing staff and other medical personnel, who may grow resilient when they are not regularly affirmed with a clinical success. Conversely, the application of very strict selection criteria may lead to excellent survival rates by a very low number of patients which also limits the total added value of ECPR within the broader perspective of OHCA [13^▪^,15^▪^]. In a recent meta-analysis, the most robust predictors for survival with a favorable neurologic outcome are a shockable rhythm and a lower serum lactate level [31]. Interestingly, Maeda *et al.* observed a very poor outcome of ECPR in patients with an initial shockable rhythm that converted to a nonshockable rhythm [32], even despite very short low-flow times. This observation was confirmed by Pozzi *et al.* who modified their institutional protocols and only provided ECPR to patients with a persisting shockable rhythm at admission. This practice led to an improvement in survival with good neurological outcome from 4.4% to 23.5% despite a low-flow time of 84 min. Simultaneously, the annual caseload dropped from 13.6 per year to 3.4 per year. Interestingly, the absolute number of survivors over two consecutive 5-year periods remained unchanged [13^▪^].

## OUTCOME PREDICTORS

In addition to the established intra-arrest characteristics that may predict outcome of ECPR, there is increasing interest in modifiable postresuscitation factors that may predict and affect outcome. The most important of these is cerebral oxygenation. Observational data suggest that cerebral oxygen saturation, measured by near-infrared spectroscopy before ECPR or shortly thereafter is higher in patients regaining consciousness after ECPR, albeit without affecting long-term neurologic outcome [33,34]. However, severe hyperoxia, which is believed to aggravate reperfusion injury was associated with poor outcome [35–38]. It has been suggested that avoiding metabolic derangement during immediate reperfusion can improve outcome [39], but more data are needed.

Apart from therapy refractory shock, cerebral injury is the most important cause of death after ECPR [40]. The timing of neuroprognostication after ECPR is subject to debate. It has been argued that neuroprognostication should not be performed within 72 h in comatose patients with referral to the guidelines of the European Resuscitation Council (ERC) [41]. Despite this, withdrawal from life-sustaining therapy occurs in more than half of nonsurviving ECPR patients [42]. It should be noted that ERC guidelines indicate that neurophysiological investigations such as Somatosensory Evoked Potentials or continuous electroencephalography can indicate poor neurological outcome with a very high sensitivity already after 24 h [43]. Therefore early prognostication such, as is common in for example the Netherlands, complies with ERC guidelines [44]. An isoelectric electroencephalogram within 24 h after admission had a sensitivity of 100% to predict mortality in a small case series [45]. There is currently also renewed interest in the prognostic value of neuro-specific enolase (NSE) to predict outcome after cardiac arrest in general and ECPR in particular, but its sensitivity as a standalone parameter is limited [45–47].

Patients with a neurological unfavorable prognosis or brain death after ECPR may become organ donors if other organ functions are preserved [48]. It even has been argued that organ donation should be incorporated in a composite endpoint when evaluating the effectiveness of resuscitation [49]. The true potential of organ donors after ECPR remains unclear and may partly be dependent on nonpatient-related factors such as consent rates and acceptance of donation after cardiac death [44,48,50].

## FUTURE PERSPECTIVE

Currently, there is only one ongoing randomized controlled trial addressing the clinical benefit of ECPR, in particular in the context of prehospital ECPR employing helicopter emergency medical services (NCT04620070). Therefore, in the foreseeable future, no new level I evidence is to be expected that will further expand the evidence concerning the benefit of in-hospital ECPR for OHCA. A meta-analysis of randomized and propensity-matched data that was performed before publication of the INCEPTION trial concluded that extracorporeal cardiopulmonary resuscitation can increase survival and favorable neurological outcome in patients with refractory out-of-hospital cardiac arrest [51]. A pooled analysis of the ARREST trial and Prague OHCA study, as well as a very recent meta-analysis of randomized controlled trials that also included the INCEPTION, resulted in the same conclusion [9,52], yet another recent meta-analysis stated that ECPR for OHCA warrants further research [53]. Although new meta-analyses addressing the clinical effectiveness of ECPR for OHCA are to be expected (e.g. CRD4202340221, CRD42023409489, CRD42023409489, CRD42023396567, CRD42023394128), they will probably provide little novel data. Meta-analyses run the risk to overlook important methodological differences between trials [54]. It may be more important to understand the relation between the differences in design and outcome of the different trials than to generate a dichotomous answer to the question of whether ECPR works or not [55]. To find definitive answers on the widespread implementability of ECPR more randomized controlled trials may be necessary [56]. It has been suggested that future multicenter randomized trials should be performed in centers with matured ECPR programs [6^▪▪^]. However, such a selection criterion imposes selection bias, when centers that failed to successfully implement an effective ECPR program are excluded. This may be overcome by selecting ECMO-ready centers without an active ECPR program who are willing to go through a structured time-limited implementation program before start of enrollment. In addition, sample size should be adequate to detect a clinically important difference [44,57]. A major issue for future research in EPCR for OHCA may be the fact that some may question whether there is still clinical equipoise between CCPR and ECPR [55,58] which is a prerequisite to justify randomization in this field [59].

## CONCLUSION

In highly-dedicated mature systems, ECPR can be an effective treatment that improves survival for patients in refractory OHCA. The ability to develop into such a system depends on several factors, including caseload and financial investment. The question remains whether ECPR for OHCA can develop into a commonplace effective treatment that can be sustainably implemented.

## Acknowledgements

*None.*

### Financial support and sponsorship

*The INCEPTION trial was funded by the Netherlands Organization for Health Research and Development and Maquet Cardiopulmonary [Getinge].*

### Conflicts of interest

*The authors of this paper were members of the Trial Steering Committee of the INCEPTION trial.*

## REFERENCES AND RECOMMENDED READING

Papers of particular interest, published within the annual period of review, have been highlighted as:

▪ of special interest
▪▪ of outstanding interest

## References

[1] Tsao CW Aday AW Almarzooq ZI. Heart disease and stroke statistics—2023 update: a report from the American Heart Association. Circulation 2023; 147:e93–e621. 36695182 10.1161/CIR.0000000000001123 PMC12135016

[2] Nishiyama C Kiguchi T Okubo M. Three-year trends in out-of-hospital cardiac arrest across the world: second report from the International Liaison Committee on Resuscitation (ILCOR). Resuscitation 2023; 186:109757. 36868553 10.1016/j.resuscitation.2023.109757

[3] Grubic N Peng YP Walker M. Bystander-initiated cardiopulmonary resuscitation and automated external defibrillator use after out-of-hospital cardiac arrest: Uncovering disparities in care and survival across the urban–rural spectrum. Resuscitation 2022; 175:150–158. 35469933 10.1016/j.resuscitation.2022.04.014

[4] Mandigers L Boersma E den Uil CA. Systematic review and meta-analysis comparing low-flow duration of extracorporeal and conventional cardiopulmonary resuscitation. Interact Cardiovasc Thorac Surg 2022; 35:ivac219. 36000900 10.1093/icvts/ivac219 PMC9491846

[5] Inoue A Hifumi T Sakamoto T. Extracorporeal cardiopulmonary resuscitation in adult patients with out-of-hospital cardiac arrest: a retrospective large cohort multicenter study in Japan. Crit Care 2022; 26:129. 35534870 10.1186/s13054-022-03998-y PMC9088043

[6▪▪] Yannopoulos D Bartos J Raveendran G. Advanced reperfusion strategies for patients with out-of-hospital cardiac arrest and refractory ventricular fibrillation (ARREST): a phase 2, single centre, open-label, randomised controlled trial. Lancet 2020; 396:1807–1816. 33197396 10.1016/S0140-6736(20)32338-2 PMC7856571

[7▪▪] Belohlavek J Smalcova J Rob D. Effect of intra-arrest transport, extracorporeal cardiopulmonary resuscitation, and immediate invasive assessment and treatment on functional neurologic outcome in refractory out-of-hospital cardiac arrest. J Am Med Assoc 2022; 327:737–747. 10.1001/jama.2022.1025 PMC8864504 35191923

[8] Havranek S Fingrova Z Rob D. Initial rhythm and survival in refractory out-of-hospital cardiac arrest. Posthoc analysis of the Prague OHCA randomized trial. Resuscitation 2022; 181:289–296. 36243225 10.1016/j.resuscitation.2022.10.006

[9] Belohlavek J Yannopoulos D Smalcova J. Intraarrest transport, extracoporeal resuscitation, and early invasive managment in refractory out-of-hospital cardiac arrest: an individual patient data pooled analysis of two randomised trials. eClinicalMedicine 2023; 59:101988. 37197707 10.1016/j.eclinm.2023.101988 PMC10184044

[10] Bol ME Suverein MM Lorusso R. Early initiation of extracorporeal life support in refractory out-of-hospital cardiac arrest: design and rationale of the INCEPTION trial. Am Heart J 2019; 210:58–68. 30738245 10.1016/j.ahj.2018.12.008

[11▪▪] Suverein MM Delnoij TSR Lorusso R. Early extracorporeal CPR for refractory out-of-hospital cardiac arrest. N Engl J Med 2023; 388:299–309. 36720132 10.1056/NEJMoa2204511

[12] Djordjevic I Gaisendrees C Adler C. Extracorporeal cardiopulmonary resuscitation for out-of-hospital cardiac arrest: first results and outcomes of a newly established ECPR program in a large population area. Perfusion 2022; 37:249–256. 33626985 10.1177/0267659121995995

[13▪] Pozzi M Grinberg D Armoiry X. Impact of a modified institutional protocol on outcomes after extracorporeal cardiopulmonary resuscitation for refractory out-of-hospital cardiac arrest. J Cardiothorac Vasc Anesth 2022; 36:1670–1677. 34130897 10.1053/j.jvca.2021.05.034

[14] Read AC Morgan S Reynolds C. The effect of a structured ECPR protocol aided by specific simulation training in a quaternary ECMO centre: a retrospective prepost study. Resusc plus 2022; 10:100234. 35509680 10.1016/j.resplu.2022.100234 PMC9059074

[15▪] Bosson N Kazan C Sanko S. Implementation of a regional extracorporeal membrane oxygenation program for refractory ventricular fibrillation out-of-hospital cardiac arrest. Resuscitation 2023; 187:109711. 36720300 10.1016/j.resuscitation.2023.109711

[16] Ottolina D Colombo R Fossali T. The efficacy of venous-arterial membrane oxygenation for emergency extracorporeal life support: results from a single-center large series over 6 years. Intern Emerg Med 2023; 18:897–906. 36961606 10.1007/s11739-023-03198-8

[17] Thevathasan T Kenny MA Krause FJ. Left-ventricular unloading in extracorporeal cardiopulmonary resuscitation due to acute myocardial infarction – a multicenter study. Resuscitation 2023; 186:109775. 36958632 10.1016/j.resuscitation.2023.109775

[18] Suverein MM Delnoij TSR Lorusso R. National and reporting differences of prehospital factors in extracorporeal cardiopulmonary resuscitation studies. Netherlands J Crit Care 2020; 28:154–161.

[19] Abrams D MacLaren G Lorusso R. Extracorporeal cardiopulmonary resuscitation in adults: evidence and implications. Intensive Care Med 2021; 48:1–15. 34505911 10.1007/s00134-021-06514-y PMC8429884

[20] Holmberg MJ Granfeldt A Guerguerian A-M. Extracorporeal cardiopulmonary resuscitation for cardiac arrest: an updated systematic review. Resuscitation 2023; 182:109665. 36521684 10.1016/j.resuscitation.2022.12.003

[21] Addison D Cheng E Forrest P. Cost-effectiveness of extracorporeal cardiopulmonary resuscitation for adult out-of-hospital cardiac arrest: a systematic review. Resuscitation 2022; 178:19–25. 35835249 10.1016/j.resuscitation.2022.07.010

[22] Doan TN Rashford S Pincus J Bosley E. Cost-effectiveness of extracorporeal cardiopulmonary resuscitation for refractory out-of-hospital cardiac arrest: a modelling study. Resusc plus 2022; 12:100309. 36187433 10.1016/j.resplu.2022.100309 PMC9515594

[23] Al-Badriyeh D Hssain AA Abushanab D. Cost-effectiveness analysis of out-of-hospital versus in-hospital extracorporeal cardiopulmonary resuscitation for out-hospital refractory cardiac arrest. Curr Probl Cardiol 2022; 47:101387. 36070844 10.1016/j.cpcardiol.2022.101387

[24] Varner-Perez SE Mathis KAL Banks SK. A descriptive study of the multidisciplinary healthcare experiences of inpatient resuscitation events. Resusc plus 2023; 13:100349. 36654725 10.1016/j.resplu.2022.100349 PMC9841215

[25] Tonna JE Selzman CH Bartos JA. The association of modifiable postresuscitation management and annual case volume with survival after extracorporeal cardiopulmonary resuscitation. Crit care Explor 2022; 4:e0733. 35923595 10.1097/CCE.0000000000000733 PMC9324623

[26] Gould J Goldstein J Travers AH. Potential candidates for emergency department initiated extracorporeal cardiopulmonary resuscitation (ECPR) in a Canadian institution. Cureus 2022; 14:e29318. 36277569 10.7759/cureus.29318 PMC9580229

[27] Gottula AL Shaw CR Gorder KL. Eligibility of out-of-hospital cardiac arrest patients for extracorporeal cardiopulmonary resuscitation in the United States: a geographic information system model. Resuscitation 2022; 180:111–120. 36183812 10.1016/j.resuscitation.2022.09.017

[28] Chandru P Mitra TP Dhanekula ND. Out of hospital cardiac arrest in Western Sydney—an analysis of outcomes and estimation of future eCPR eligibility. BMC Emerg Med 2022; 22:31. 35227204 10.1186/s12873-022-00587-8 PMC8887068

[29] Pudil J Rob D Smalcova J. Pulmonary embolism related refractory out-of-hospital cardiac arrest and extracorporeal cardiopulmonary resuscitation: Prague OHCA study posthoc analysis. Eur Hear J Acute Cardiovasc Care 2023; 12:507–512. 10.1093/ehjacc/zuad052 PMC10449371 37172033

[30] Bartos JA Yannopoulos D. Starting an Extracorporeal cardiopulmonary resuscitation program: success is in the details. Resuscitation 2023; 187:109792. 37044354 10.1016/j.resuscitation.2023.109792

[31] Bertic M Worme M Foroutan F. Predictors of survival and favorable neurologic outcome in patients treated with eCPR: a systematic review and meta-analysis. J Cardiovasc Transl Res 2022; 15:279–290. 35194733 10.1007/s12265-021-10195-9

[32] Sakamoto T Morimura N Nagao K. Extracorporeal cardiopulmonary resuscitation versus conventional cardiopulmonary resuscitation in adults with out-of-hospital cardiac arrest: a prospective observational study. Resuscitation 2014; 85:762–768. 24530251 10.1016/j.resuscitation.2014.01.031

[33] Mandigers L den Uil CA Belliato M. Higher mean cerebral oxygen saturation shortly after extracorporeal cardiopulmonary resuscitation in patients who regain consciousness. Artif Organs 2023; 47:1479–1489. 37042484 10.1111/aor.14548

[34] Bertini P Marabotti A Paternoster G. Regional cerebral oxygen saturation to predict favorable outcome in extracorporeal cardiopulmonary resuscitation: a systematic review and meta-analysis. J Cardiothorac Vasc Anesth 2023; 37:1265–1272. 36759264 10.1053/j.jvca.2023.01.007

[35] Hong S Jang JH Yang JH. Optimal arterial blood gas tensions for the prognosis of favorable neurological outcomes in survivors after extracorporeal cardiopulmonary resuscitation. J Clin Med 2022; 11:4211. 35887974 10.3390/jcm11144211 PMC9323021

[36] Shou BL Ong CS Premraj L. Arterial oxygen and carbon dioxide tension and acute brain injury in extracorporeal cardiopulmonary resuscitation patients: analysis of the extracorporeal life support organization registry. J Hear Lung Transplant 2023; 42:503–511. 10.1016/j.healun.2022.10.019 PMC10050131 36435686

[37] Kashiura M Yasuda H Kishihara Y. Association between short-term neurological outcomes and extreme hyperoxia in patients with out-of-hospital cardiac arrest who underwent extracorporeal cardiopulmonary resuscitation: a retrospective observational study from a multicenter registry. BMC Cardiovasc Disord 2022; 22:163. 35410132 10.1186/s12872-022-02598-6 PMC9003952

[38] Stoll SE Paul E Pilcher D. Hyperoxia and mortality in conventional versus extracorporeal cardiopulmonary resuscitation. J Crit Care 2022; 69:154001. 35217372 10.1016/j.jcrc.2022.154001

[39] Philipp A Pooth J-S Benk C. Enabling the control of reperfusion parameters in out-of-hospital cardiac arrest: first applications of the CARL system. Perfusion 2023; 38:436–439. 36416680 10.1177/02676591221141325 PMC9932608

[40] Zotzmann V Lang C Bemtgen X. Mode of death after extracorporeal cardiopulmonary resuscitation. Membranes (Basel) 2021; 11:270. 33917888 10.3390/membranes11040270 PMC8068242

[41] Teixeira JP Kraai E Wray TC. Extracorporeal CPR for out-of-hospital cardiac arrest. N Engl J Med 2023; 388:1914–1915. 10.1056/NEJMc2302405 37195954

[42] Carlson JM Etchill E Whitman G. Early withdrawal of life sustaining therapy in extracorporeal cardiopulmonary resuscitation (ECPR): results from the Extracorporeal Life Support Organization registry. Resuscitation 2022; 179:71–77. 35934132 10.1016/j.resuscitation.2022.07.038 PMC9530029

[43] Nolan JP Sandroni C Böttiger BW. European Resuscitation Council and European Society of Intensive Care Medicine guidelines 2021: postresuscitation care. Intensive Care Med 2021; 47:369–421. 33765189 10.1007/s00134-021-06368-4 PMC7993077

[44] Suverein MM Lorusso R van de Poll MCG. Extracorporeal CPR for out-of-hospital cardiac arrest. N Engl J Med 2023; 388:1916–1917. 37195957 10.1056/NEJMc2302405

[45] Bartos JA Nutting L Carlson C. Abstract 10: early neuroprognostication after refractory VF/VT cardiac arrest requiring ECPR. Circulation 2020; 2:e0214.

[46] Haertel F Babst J Bruening C. Effect of hemolysis regarding the characterization and prognostic relevance of neuron specific enolase (NSE) after cardiopulmonary resuscitation with extracorporeal circulation (eCPR). J Clin Med 2023; 12:3015. 37109353 10.3390/jcm12083015 PMC10146981

[47] Kim HB Yang JH Lee YH. Are serial neuron-specific enolase levels associated with neurologic outcome of ECPR patients: a retrospective multicenter observational study. Am J Emerg Med 2023; 69:58–64. 37060630 10.1016/j.ajem.2023.03.047

[48] Fainberg NA Morrison WE West S. Organ donation from patients on extracorporeal membrane oxygenation at the time of death. Crit Care Explor 2022; 4:e0812. 36567782 10.1097/CCE.0000000000000812 PMC9760628

[49] Achana F Petrou S Madan J. Cost-effectiveness of adrenaline for out-of-hospital cardiac arrest. Crit Care 2020; 24:579. 32981529 10.1186/s13054-020-03271-0 PMC7520962

[50] Manintveld OC Roest S Taverne YJHJ. Extracorporeal CPR for out-of-hospital cardiac arrest. N Engl J Med 2023; 388:1913–1914. 37195952 10.1056/NEJMc2302405

[51] Scquizzato T Bonaccorso A Consonni M. Extracorporeal cardiopulmonary resuscitation for out-of-hospital cardiac arrest: a systematic review and meta-analysis of randomized and propensity score-matched studies. Artif Organs 2022; 46:755–762. 35199375 10.1111/aor.14205 PMC9307006

[52] Scquizzato T Bonaccorso A Swol J. Refractory out-of-hospital cardiac arrest and extracorporeal cardiopulmonary resuscitation: a meta-analysis of randomized trials. Artif Organs 2023; 47:806–816. 36929354 10.1111/aor.14516

[53] Low CJW Ramanathan K Ling RR. Extracorporeal cardiopulmonary resuscitation versus conventional cardiopulmonary resuscitation in adults with cardiac arrest: a comparative meta-analysis and trial sequential analysis. Lancet Respir Med 2023; 11:883–893. 37230097 10.1016/S2213-2600(23)00137-6

[54] Mbuagbaw L Aves T. Meta-analysis of pragmatic and explanatory trials 2022; 2345:147–158. 10.1007/978-1-0716-1566-9_9 34550589

[55] Scquizzato T Yannopoulos D Belohlavek J. Extracorporeal CPR after the INCEPTION trial: no one steps twice into the same river. Artif Organs 2023; 47:802–805. 37171146 10.1111/aor.14520

[56] Keaney JFJ Münzel T. Extracorporeal CPR in out-of-hospital cardiac arrest – still on life support? N Engl J Med 2023; 388:370–371. 36720138 10.1056/NEJMe2214116

[57] Cho S-M Geocadin R Whitman GJ. Extracorporeal CPR for out-of-hospital cardiac arrest. N Engl J Med 2023; 388:1915–1916. 10.1056/NEJMc2302405 PMC10564385 37195956

[58] Tonna JE Keenan HT Weir C. A qualitative analysis of physician decision making in the use of extracorporeal cardiopulmonary resuscitation for refractory cardiac arrest. Resusc Plus 2022; 11:100278. 35898590 10.1016/j.resplu.2022.100278 PMC9309663

[59] Suverein MM Shaw D Lorusso R. Ethics of ECPR research. Resuscitation 2021; 169:136–142. 34411691 10.1016/j.resuscitation.2021.08.007

